# Approaching In Vivo Models of Pneumococcus–Host Interaction: Insights into Surface Proteins, Capsule Production, and Extracellular Vesicles

**DOI:** 10.3390/pathogens10091098

**Published:** 2021-08-28

**Authors:** Alfonso Olaya-Abril, José A. González-Reyes, Manuel J. Rodríguez-Ortega

**Affiliations:** 1 Departamento de Bioquímica y Biología Molecular, Campus de Excelencia Internacional CeiA3, Universidad de Córdoba, 14071 Córdoba, Spain; b22olaba@uco.es; 2 Departamento de Biología Celular, Fisiología e Inmunología, Campus de Excelencia Internacional CeiA3, Universidad de Córdoba, 14071 Córdoba, Spain; bc1gorej@uco.es

**Keywords:** host-pathogen interaction, capsule, membrane vesicles, surface proteins, proteomics

## Abstract

Infections caused by the Gram-positive bacterium *Streptococcus pneumoniae* have become a major health problem worldwide because of their high morbidity and mortality rates, especially in developing countries. This microorganism colonizes the human upper respiratory tract and becomes pathogenic under certain circumstances, which are not well known. In the interaction with the host, bacterial surface structures and proteins play major roles. To gain knowledge into gradual changes and adaptive mechanisms that this pathogen undergoes from when it enters the host, we mimicked several in vivo situations representing interaction with epithelial and macrophage cells, as well as a condition of presence in blood. Then, we analyzed, in four pneumococcal strains, two major surface structures, the capsule and extracellular vesicles produced by the pneumococci, as well as surface proteins by proteomics, using the “shaving” approach, followed by LC-MS/MS. We found important differences in both surface ultrastructures and proteins among the culture conditions and strains used. Thus, this work provides insights into physiological adaptations of the pneumococcus when it interacts with the host, which may be useful for the design of strategies to combat infections caused by this pathogen.

## 1. Introduction

Infections by *Streptococcus pneumoniae* (the pneumococcus), a major agent of pneumonia worldwide, cause high rates of morbidity and mortality mostly in developing countries, with children, the elderly, and immunocompromised patients the most susceptible groups of the population [1]. In fact, around one million children <5 years old die every year because of infections caused by this pathogen [2]. Although there are capsule-based and conjugate vaccines available, which are of limited efficacy [3,4], pneumococcal diseases are also increasing in developed countries because of emerging antibiotic resistance [5], capsular switching [6], and vaccine-serotype replacement [7]. This has raised concern in public health systems, as vaccine-type strains can be replaced by more virulent and antibiotic-resistant strains, not covered by current vaccines [8].

Adaptive responses are fundamental for pathogens when interacting with the host. The pneumococcus colonizes the human nasopharynx at early ages [9]. In many cases, this bacterium coexists as a commensal in the upper respiratory tract. However, it may become pathogenic under certain circumstances, causing either non-invasive (e.g., otitis media, sinusitis, pneumonia) or invasive (e.g., bacteremia, meningitis, sepsis) disease [10]. Understanding how pathogens adapt to their host niche and can change depending on the actual host–pathogen interaction environment and respond to nutrient limitations or to the host immune system is critical to know the way in which microorganisms modulate their physiology and responses to switch from a colonizing to a pathogenic status. In this regard, comparative studies that simulate different host–interaction conditions constitute a valuable choice to gain insight into adaptive mechanisms of microbes to the environment. The microbial surface is the preferential site of interaction between cells and their environment [11] as it acts as both a barrier and the site to interplay with the host at multiple levels. In this interaction, numerous structures and molecules play major roles, being especially relevant to the capsule (which surrounds many bacterial cells), and surface proteins, which have key functions and have the highest chances to be recognized by the host immune system [12,13]. In addition, extracellular vesicles (EVs) released from the bacterial surface have been revealed as a critical way to interact with the surroundings, and in the case of pathogenic microorganisms, they play important roles during the infection process [14,15,16].

In this work, we examined the host–pathogen interaction using different culture conditions for the pneumococcus, in order to understand how the major surface-attached and surface-derived ultrastructures and proteins adapt to these environments. We analyzed, in four pneumococcal strains, the amount of capsule and EVs, as well as the surface proteins using the “shaving” approach, followed by LC-MS/MS in a condition resembling bacteremia, and in other conditions that simulate interaction with host macrophages and epithelial cells. We found strain-dependent differences in EV production and surface protein profiles, and a general increase in capsule amount in the bacteremia-like model, as well as a decrease in capsule production when the bacteria contact the host cells. Thus, this study provides new insights into adaptation in the physiology of pneumococcus when it interacts with the host.

## 2. Results

**Figure 1:**
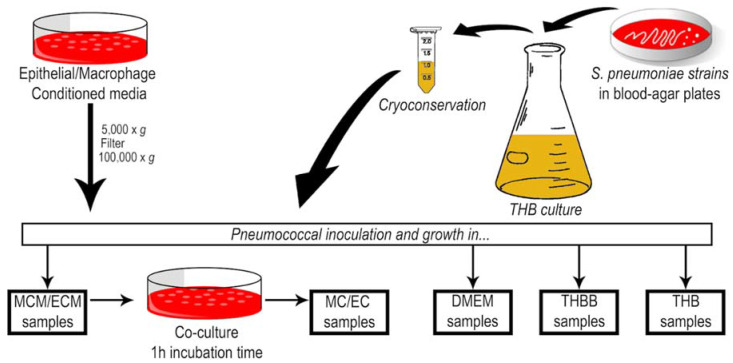
Sample preparation workflow: MCM, macrophages conditioned medium; ECM, epithelial conditioned medium; MC, co-culture with J774 macrophages (macrophages contact); EC, co-culture with A549 epithelial cells (epithelial contact); DMEM, Dulbecco’s modified Eagle’s medium; THB, Todd–Hewitt broth; THBB, Todd–Hewitt broth supplemented with 5% sheep blood.

We analyzed the capsule and EV production in different culture media and models of interaction with host cells in four pneumococcal strains (Figure 1): the avirulent and non-encapsulated R6 reference strain and three virulent strains pertaining to three capsulated serotypes: 1, 6B, and 8. First, we compared the growth of these strains in five liquid culture media: THBB as representative of a model of bacteremia, and THB as its control; and the eukaryotic cell DMEM medium conditioned either by macrophages (MCM) or by epithelial cells (ECM) as models of interaction with host cells during colonization for which DMEM was used as control.

**Figure 2:**
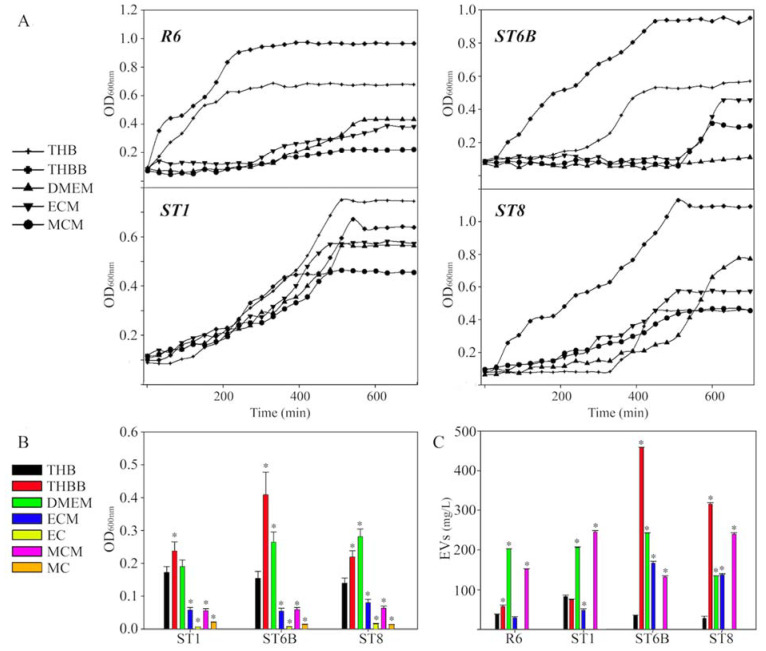
Effect of culture media on *Streptococcus pneumoniae* growth, capsule, and extracellular vesicles production: (**A**) pneumococcal growth curves in the culture media used; (**B**) capsule production measured by the stain-all assay; (**C**) production of extracellular vesicles (EVs) by pneumococcal strains per culture media. * *p* < 0.05.

Significant differences were observed in the growth curves for the tested media (Figure 2A). There was a faster growth in THBB than in THB, except for the ST1 strain. The growth in THBB was also faster than in the other media analyzed, i.e., MCM and ECM, as well as their control DMEM. In these last three media, representing direct host–pathogen interaction, R6 showed the lowest growth, while the ST1 strain exhibited the best adaptation to these media.

**Figure 3:**
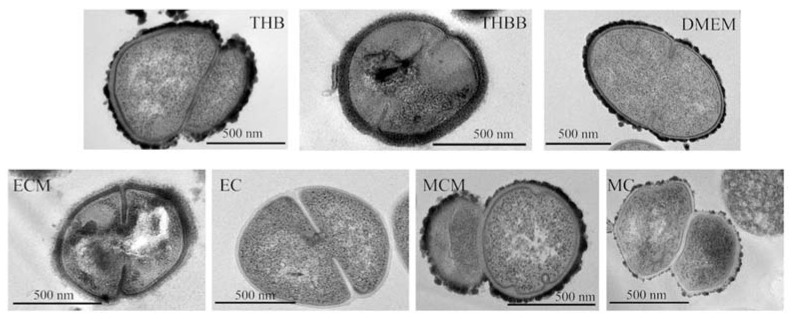
Transmission electron microscopy of serotype 1 *Streptococcus pneumoniae* showing the differential amount of capsule depending on culture media. Panels show detailed pictures of pneumococcal bacterial cells in each of the seven conditions used in this study, i.e., culture in THB medium, culture in THB with blood (THBB), cultured in DMEM medium, cultured in DMEM that was conditioned either by epithelial cells (ECMs) or macrophages (MCMs), and bacteria recovered after direct contact with either epithelial cells (ECs) or macrophages.

Next, we studied capsule production by the three encapsulated serotypes (Figure 2B). The growth in THBB caused a clear increase in the amount of capsule produced in the three strains when compared to the absence of blood, i.e., THB. However, the measured capsule clearly diminished after growing the pneumococci either in a medium previously conditioned by macrophages or epithelial cells, and a higher decrease was observed in bacterial cells recovered after direct contact with both macrophages (MC) and epithelial cells (EC) when comparing these conditions to their corresponding control DMEM. In all cases, the amount of capsule in cultured cell-conditioned media or after direct contact with cells was lower than in the bacteremia-like model of THBB. We visualized this effect by electron microscopy in the ST1 strain (Figure 3), in which a clear decrease in capsule amount was appreciated in bacterial cells in direct contact with cultured eukaryotic cells (EC and MC), compared to pneumococci cultured in an environment conditioned by the cultured cells (ECM and MCM), as well as in comparison with bacteria grown in THBB. In this case, the increase in capsule for THBB-cultured bacteria was not so evident, compared to those cultured in THB, as shown previously in the colorimetric assays (Figure 2B).

We also analyzed the amount of EVs produced by the four tested strains in the different media (Figure 2C). The effect of the culture media was not homogeneous among the strains, as the presence of blood in the medium (THBB) caused an overproduction of EVs except in the ST1 strain, when compared to its control THB. However, the exposure to cellular factors released by cultured cells was dissimilar in the four pneumococcal strains: the growth in ECM caused a decrease in pneumococcal EVs produced by three strains, but those produced by ST8 were not affected, compared to its control DMEM. On the other hand, EVs of pneumococci grown in MCM decreased in R6 and ST6B and increased in ST1 and ST8. Pneumococcal EVs in cultures after direct contact with host cells were not measured because of the technical difficulties to separate them from potential exosomes released by the eukaryotic cells.

Next, we analyzed the surface proteins of the four strains in the seven culture conditions tested in this work, using the approach of “shaving” intact living bacterial cells with trypsin, followed by LC-MS/MS analysis. In total, 279 different surface proteins were identified (Supplemental Dataset 1), of which 23 were cell-wall proteins with an LPXTG motif; another 23 proteins were classified as lipoproteins; 218 were membrane proteins, of which 69 had only 1 transmembrane domain and 149 were multi transmembrane; 15 were secreted proteins. Considering the 28 different strains and culture conditions examined, the most widely distributed proteins were those with the LPXTG cell-wall-anchoring motif: the beta-galactosidase precursor Spr0565, the ZmpB Spr0581, and the IgA1 protease Spr1042 were identified in 17, 17, and 16 samples, respectively. Additionally, the cell-wall proteins Spr0561 and Spr0328 were both identified in 13 samples. Another 2 proteins, CbpA (Spr1995), which is predicted as secreted, and PspA, a membrane protein, were identified in 15 samples. Remarkably, the prophage-encoded protein PblB was also identified in all the strains except R6, being found in all the studied conditions.

**Figure 4:**
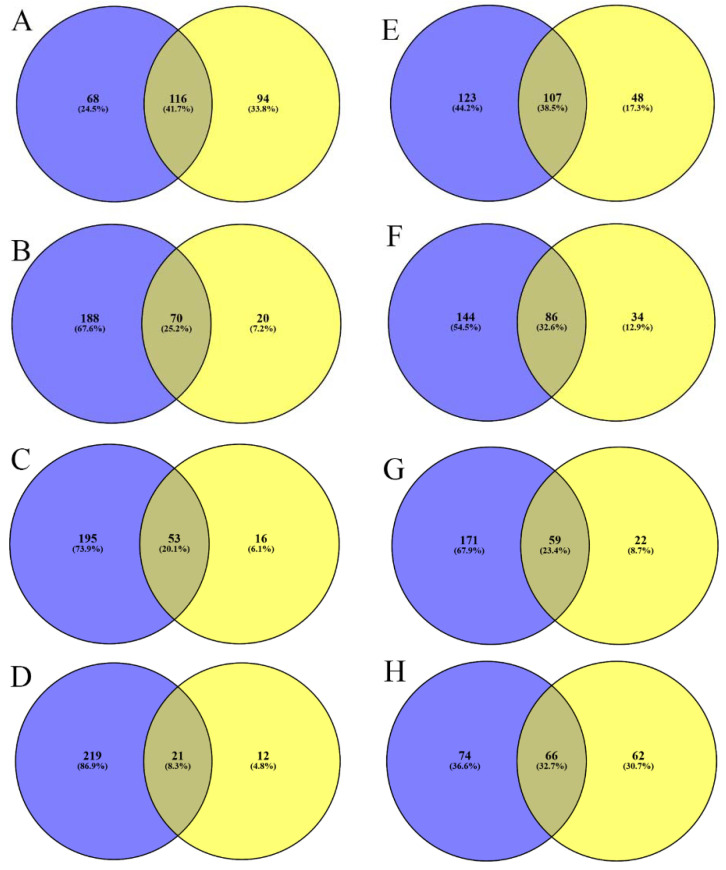
Comparison of identified surface proteins in different host-pathogen interaction models. Each Venn diagram represents the surface proteins identified in common or exclusive for each group in all the categories of interaction (as defined in the text and in Supplemental Dataset 1), with the left blue circle as the control (no interaction) and the right yellow circle as the cultures representing interaction: (**A**) “host interaction”; (**B**) “contact”, (**C**) “contact with macrophages”; (**D**) “contact with epithelial cells”; (**E**) “interaction with cells”; (**F**) “interaction with macrophages”; (**G**) “interaction with epithelial cells”; (**H**) “interaction with blood.”

In order to investigate changes in surface protein profiles, we established different comparisons based on host–pathogen interaction levels according to our proposed models (see all the comparison categories and identifications in Supplemental Dataset 1). Thus, the most general comparison was “host interaction,” using as controls THB and DMEM; in “contact,” the conditions described as “macrophage contact (MC)” and “epithelial cell contact (EC)” were compared with the rest. The comparisons for only MC and EC were also carried out. We also expanded this comparison to “interaction with cells,” which included the culture media previously conditioned by the cells. As before, this category was also subdivided to compare only interaction with macrophages or with epithelial cells. Finally, we analyzed the model of bacteremia, comparing THBB with its control THB. Figure 4 summarizes the proteins identified exclusively or in common for each category, in all the comparisons performed. Although the changes in protein profiles were profuse, we highlight that, in the model of “epithelial cells contact,” there was a generalized disappearance of most cell wall proteins, as well as the transmembrane hypothetical protein Spr1584.

**Figure 5:**
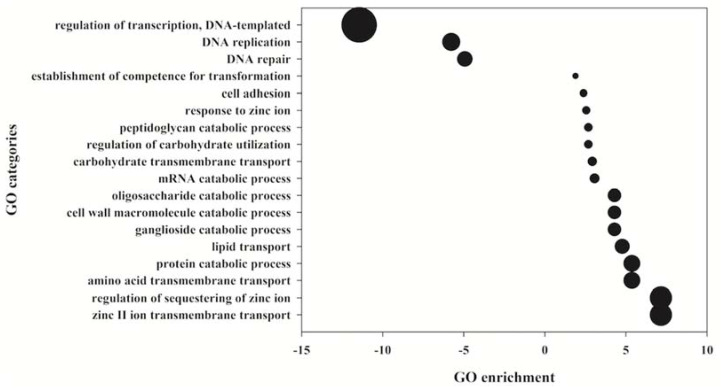
Gene ontology groups affected by *Streptococcus pneumoniae* after the contact with epithelial cells. Only GO groups (biological function, third level) that change significantly, with a *p*-value < 0.05 after a hypergeometric distribution analysis (E(GOi)) are shown as the actual sample enrichment (ASE)/expected sample enrichment (ESE) ratio. If the ratio was lower than 1 (over-represented in the ESE), the inverse value with a negative symbol is shown. The size of the bubble corresponds to the absolute value of the ASE/ESE or ESE/ASE ratio. The whole genome of *S. pneumoniae* R6 was used as reference. The GO enrichment parameter E(GOi) was calculated by the formula: (E(GOi)) = sample size/genome size × GOi.

A gene ontology (GO) enrichment analysis of this model performed with all the identified proteins (including also the cytoplasmic proteins, removed from the categories of surface proteins) revealed, among others, a clear enrichment of the GO terms “cell adhesion,” as well as other related to zinc ion binding or transport (“response to zinc ion”, “regulation of sequestering of zinc ion”, and “zinc II ion transmembrane transport”) (Figure 5).

Likewise, two membrane proteins (Spr0343 and Spr1333) appeared after cell interaction. It is noteworthy that the predicted secreted protein Spr2021, also known as PcsB, was identified only in two strains, R6 and ST6B, and only in THB and THBB but not in the conditions simulating contact with cells.

## 3. Discussion

The purpose of this work consisted of providing insights into the pneumococcal surface structure and protein variations when comparing different culture conditions resembling in vivo models of infection. The capsule is a widely studied and characterized structure of the pneumococcal surface, which acts also as a major virulence factor [17,18]. Recently, the production of EVs by this microorganism has been described. These structures are immunoreactive and protective against infection [14], in addition to having an immunomodulatory effect [19]. The set of surface proteins, known as the “surfome,” has also been described in collections of clinical isolates to define potential candidates for vaccines and diagnostics [20,21]. However, there is a lack of studies focusing on how capsule, EVs, and surface proteins vary when the pathogen interacts with the host. For this purpose, we defined five models of host–pathogen interaction, which, although with limitations since they are not real in vivo infections, can help to understand the physiological adaptation of the pneumococcus when it contacts the host. One model simulates bacteremia (THBB); two simulate the pneumococcus in an environment close to target cells (represented by DMEM conditioned by macrophages or epithelial cells, i.e., MCM and ECM), and the other two simulate the bacteria in direct contact with host cells (MC and EC). We used THB as the control for the first model and DMEM as the control for the other four models. In the bacteremia model, all the strains except ST1 grew much faster in THBB, compared to the other media. It is evident that this difference is due to the presence of blood, as the growth in THB was slower. However, we also recognize that this model might be somehow far from a real bacteremia situation, which is defined as the presence of bacteria in the bloodstream. Using a DMEM-based medium containing blood could have been an alternative model, but even in such a medium, the bacteremia is not completely emulated.

We chose three virulent strains belonging to three representative serotypes: ST1, ST6B, and ST8. ST1 is highly invasive and, although included in conjugate vaccines, has been highly prevalent among the circulating isolates in Spanish elderly people in the last decade [22]. ST6B has been prevalent in children <5 years old [23,24]. Finally, ST8 is also highly invasive and is not included in current conjugate vaccines. This serotype has emerged as a replacement serotype [25] and displays epidemic potential [22,26].

In this work, we found that the three capsulated strains produced the highest amount of capsule in the medium with blood (THBB) and that the capsule decreased after contacting with released factors from host cells (MCM, ECM) and even more after direct contact with the cells (MC, EC). A possible explanation is that when the pneumococcus circulates in the blood in a septicemic episode, it synthesizes a thick capsule to avoid or minimize opsonization from blood antibodies [17,27]. When it contacts the host cells, the capsule must be thinner to allow adhesin proteins to adhere, as observed in biofilms [28]. The effect of growth medium on EV production, however, was not homogeneous, as ST6B and ST8 strains, but neither R6 nor ST1, underwent an overproduction in THBB. Additionally, contact with host-cell-released factors caused an increase in EVs produced by some strains but not by others. However, it can be observed that a lower amount of capsule is correlated with a higher production of EVs, as the capsule may hinder the release of vesicles.

“Shaving” live cells with proteases coupled to LC-MS/MS analysis is a powerful approach to identify in a fast and reliable way the most abundant and exposed surface proteins, a fraction known as the “surfome” or “surfaceome” [13]. Originally described for *Streptococcus pyogenes* [29], it has been applied to numerous microorganisms and parasites. We have extensively applied this strategy in *S. pneumoniae* [20,21,30] and *S. suis* [31,32,33,34,35] aiming at discovering potential vaccine and diagnostics candidates. Nevertheless, thus far, we have always used bacteria cultured in standard laboratory liquid broths, which might not be representative of what is really expressed in vivo. For this reason, we compared different culture models that might emulate diverse in vivo conditions. Ours is a comprehensive work integrating studies on surface proteins using proteomics, as well as capsule and EV production in different culture conditions.

Our surfomic analysis identified a total of 279 surface proteins. Those anchored to the cell wall were among the most frequently identified and widely distributed within the four strains and culture conditions, because of their relative abundance in bacterial surfaces, as already described in numerous works using this “shaving” approach [36,37,38,39]. The purpose of this work was not to validate all the changes at the surfomic level in all the conditions and strains analyzed but to provide insights into possible tracks to be explored in future investigations, as many changes in individual proteins were detected. Indeed, more general changes in a group or category of proteins could indicate a more specific physiological change or adaptation. In this regard, the finding that most of the identified cell-wall proteins tended to disappear after contact with epithelial cells is especially interesting, although the meaning of this change remains unclear. It is also very noteworthy that PcsB, annotated in R6 as Spr2021 and referenced in this work with this locus name, was only identified in the strains R6 and ST6B, both in THB and THBB but not after contact with cultured cells or their released factors. PcsB is a major and immunogenic protein [40] that is widely expressed in pneumococcus. Although it is predicted as secreted, it has been demonstrated that the preprotein form localizes to the plasma membrane [41]. In fact, we have identified it using the “shaving” approach in many pneumococcal strains cultured in complex broths and chemically defined media [20,21,30]. In a more recent study, we have shown also that this protein decreases in an iron deprivation condition, which simulates what occurs in vivo [42]. The concentrations of divalent metal cations in vivo are in general quite low, which explains the increase in genes encoding for functions related to metal cation binding and transport. In fact, our present study shows that GO categories related to zinc binding and transport are enriched when the pneumococcus contacts epithelial cells, thus revealing that, compared to a planktonic culture condition, the bacteria presumably have lower metal cation levels available and have to synthesize more transporters and binding proteins to uptake such ions.

It is noteworthy that we also identified the prophage-encoded PblB in nine out of the 28 combinations of this study (four strains, seven culture conditions each), in three out of the four strains analyzed. This protein was first described as a platelet-binding and activator in *S. mitis* [43,44]. In the pneumococcus, PblB possesses a galactose-binding domain that mediates adhesion to host epithelial cells [45]. We previously identified this protein in the “surfome” of approximately two-thirds of the clinical isolates of a previous study using the “shaving” approach. Moreover, we have demonstrated that this protein is recognized by human sera and showed the highest discrimination capacity between pneumococcus-infected children and controls in a protein chip array platform [21]. Additionally, a positive correlation has been reported between the presence of this protein and mortality in patients with invasive pneumococcal disease (IPD) [46,47].

In conclusion, this work shows evidence of gradual changes in the surface ultrastructures and surface proteins of the pneumococcus from a more planktonic to a more host-interacting model, using conditions that resemble different stages of host–pathogen interactions, even though the models used in this study might not totally resemble what really happens in vivo. The capsule shows its maximum thickness in a model resembling bacteremia and decreases when the pathogen contacts host cell surfaces. Surface proteins also adapt to the medium. Production of EVs varies according to the strain and infection model. Future strategies to study host–pathogen interactions should incorporate a global analysis of surface structures and proteins in different models to gain insight into changes evolving from non-interacting to a more pathogenic status of the microorganisms, in order to design more effective strategies to fight against bacterial infections.

## 4. Materials and Methods

### 4.1. Cell Lines, Bacterial Strains, and Growth

J774 macrophages and A549 epithelial cells were cultured at 37 °C in a 5% CO_2_ atmosphere in the air, in Dulbecco’s modified Eagle’s medium (DMEM) supplemented with 10% fetal bovine serum, 10% NCTC, and 1% non-essential amino acids. Conditioned media from macrophages and epithelial cells (macrophages-conditioned media (MCM); epithelial cells-conditioned media (ECM)) were recovered as supernatant, centrifuged at 800× *g* for 3 min, filtered with 0.22 µm pore-size filters, centrifuged at 100,000× *g* and stored at −20 °C until use. *Streptococcus pneumoniae* strains (R6, serotype 2; ST1, serotype 1; ST6B, serotype 6B; ST8, serotype 8) were grown at 37 °C in different media: Todd–Hewitt broth (THB); THB supplemented with 5% sheep blood (THBB) lysed with distilled water, centrifuged to remove cell debris and filtered with 0.22 µm pore-size filters; DMEM; MCM; ECM; in co-culture with macrophages (MC) or with epithelial cells (EC) during 1 h of incubation. In this case, pneumococci were harvested by centrifugation, first at 800× *g* for 3 min, to remove eukaryotic cell debris, and later at 5000× *g* for 10 min, checking the absence of eukaryotic cells by optical microscopy visualization.

### 4.2. “Shaving” of Pneumococcal Living Cells

Pneumococcal strains were “shaved” for surface protein identification, as already described [20,21,30], but with some modifications. Briefly, 10 mL of each strain were grown in the corresponding media (THB, THBB, DMEM, MCM, or ECM) to an OD_600_ = 0.3, which corresponds to approximately 10^8^ bacterial cells/mL. When growth did not reach the OD, the cultures were diluted to the lowest OD value for each individual strain. In the case of bacteria in contact with eukaryotic cells, the bacterial cells were resuspended in a volume of DMEM yielding the lowest OD for each individual strain. Under the growth conditions tested, equal volumes of each strain were further processed in order to normalize the number of bacterial cells. Afterward, the bacteria were pelleted by centrifugation at 3500 × *g* for 10 min. Bacterial pellets were washed twice with PBS, resuspended in 1 mL of PBS containing 30% sucrose (pH 7.4), and digested with 5 µg trypsin (Promega, Madison, WI) for 30 min at 37 °C. The resulting digestion mixtures were redigested with 2 µg trypsin overnight at 37 °C. Samples were cleaned using Oasis HLB extraction cartridges (Waters, Milford, MA, USA) as described [48].

### 4.3. LC-MS/MS Analysis

All analyses were performed with a Surveyor HPLC System in tandem with an LTQ-Orbitrap mass spectrometer (Thermo Fisher Scientific, San Jose, CA, USA) equipped with a nanoelectrospray ionization interface (nESI). The separation column was 150 mm × 0.150 mm ProteoPep2 C18 (New Objective, MA, USA) at a post-split flow rate of 1 μL/min. For trapping of the digest, a 5 mm × 0.3 mm precolumn Zorbax 300 SB-C18 (Agilent Technologies, Germany) was used. One-fourth of the total sample volume, i.e., 5 μL, was trapped at a flow rate of 10 μL/min for 10 min and 5% acetonitrile/0.1% formic acid. Afterward, the trapping column was switched online with the separation column, and the gradient was started. Peptides were eluted with a 60 min gradient of 5–40% of acetonitrile/0.1% formic acid solution at a 250 nL/min flow rate. All separations were performed using a gradient of 5–40% solvent B for 60 min. MS data (Full Scan) were acquired in the positive ion mode over the 400–1500 m/z range. MS/MS data were acquired in a dependent scan mode, selecting automatically the five most intense ions for fragmentation, with dynamic exclusion set to on. In all cases, a nESI spray voltage of 1.9 kV was used.

### 4.4. Protein Identification by Database Searching

Tandem mass spectra were extracted using Proteome Discoverer 1.0 (Thermo Fisher Scientific). All MS/MS samples were analyzed using Sequest (Thermo Fisher Scientific, version v.27), applying the following search parameters: peptide tolerance, 10 ppm; tolerance for fragment ions, 0.8 Da; b- and y-ion series; oxidation of methionine and deamidation of asparagine and glutamine were considered as variable modifications; maximum trypsin missed cleavage sites, 3. The raw data were searched against an in-house joint database containing the protein sequences from all the sequenced and annotated *S. pneumoniae* strains available at the NCBI ftp site. Peptide identifications were accepted if they exceeded the filter parameter Xcorr score vs. charge state with SequestNode Probability Score (+1 = 1.5, +2 = 2.0, +3 = 2.25, +4 = 2.5). With these search and filter parameters, no false-positive hits were obtained. For proteins identified from only one peptide, fragmentations were checked manually. Strain R6 was used as a reference for providing the accession numbers of the identified proteins; whenever a protein belonging to another strain was found, homology with a corresponding protein of strain R6 was given by using protein-BLAST. If homology with R6 proteins was not observed, then the protein accession numbers of the other strains were used. Primary predictions of subcellular localization were assigned by using the LocateP web-based algorithm [49].

### 4.5. Pneumococcal EVs Production and Quantification

EVs were isolated as described [14], by using a series of Optiprep gradient layers with concentrations ranging from 35–5% (*w/v*). Briefly, cells at different ODs were pelleted from 1 L cultures and the supernatants were filtered through a 0.22 μm pore size filter (Millipore, Burlington, MA, USA). The supernatants were then centrifuged at 100,000 × *g* for 1.5 h at 4 °C to sediment the vesicular fraction. The pellets were mixed with 2 mL of Optiprep solution (Sigma-Aldrich, St. Louis, MO, USA), yielding 35% (*w/v*) Optiprep final concentration. The crude vesicle sample was then overlaid with a series of Optiprep gradient layers with concentrations ranging from 35% to 5% (*w/v*). The gradients were centrifuged (100,000 × g, 16 h), and 1 mL fractions were removed from the top. The fractions were then centrifuged at 100,000 × *g* for 1 h at 4 °C and recovered. Finally, vesicles were air-dried, weighed, and resuspended in 1 mL PBS.

### 4.6. Capsule Quantification

The amount of capsule was determined using the stains-all assay (Sigma) for detecting acidic polysaccharides, as described [50]. The bacteria were cultured to late-exponential phase, then 5 mL was centrifuged for 10 min at 5000× *g*, washed with PBS, and resuspended in 0.5 mL 0.85% NaCl. A total of 10 μL were removed to make dilutions in PBS for plating out to quantify the number of bacteria. To the remaining bacterial suspension, 2 mL of a solution containing 20 mg 1-ethyl-2(3-(1-ethylnaphthho- (1,2-d)thiazolin-2-ylidene)-2methylpropenyl)naphthho-(1,2d)thiazoliumbromide (stains-all) and 60 mL glacial acetic acid in 100 mL 50% formamide was added, and the OD_640_ determined; 0.5 mL NaCl with 2 mL stains-all solution was used as a blank.

### 4.7. Electron Microscopy

The pneumococcal capsule was observed by transmission electron microscopy as described [50,51] but without lysin or acetate in the cacodylate buffer. Bacteria were harvested by centrifugation for 10 min at 5000× *g*, then washed twice, resuspended in PBS, and fixed with 2% paraformaldehyde and 2.5% glutaraldehyde in 0.1 M cacodylate buffer (pH 7) with 0.075% ruthenium red for 20 min on ice. The samples were fixed again with the fixing solution for 3 h, washed with cacodylate buffer containing 0.075% ruthenium red, and then post-fixed with 1% osmium tetroxide in cacodylate buffer containing 0.075% ruthenium red for 1 h at room temperature. After dehydration in an ascendant series of ethanol, the pieces were transferred to propylene oxide and sequentially infiltrated in EMbed 812 resin (EMS, USA). Blocks were formed in fresh resin that was allowed to polymerize for 48 h at 65 °C. Blocks were cut in an Ultracut Reicher ultramicrotome to obtain 40–60 nm width sections using a diamond knife. The sections were observed and photographed in a Jeol JEM 1400 Transmission Electron Microscope.

### 4.8. Statistics and Data Analysis

Statistical analyses were performed using SPSS v 21.0.0.0. Student’s *t*-test (2-tailed) was applied for experiments involving pairwise comparisons. *p* < 0.05 was considered significant. The BLAST2GO platform was used to assign gene ontology annotations to gene products [52]. Gene ontology analysis was performed using the web-based algorithm ComparativeGO [53].

## Data Availability

The proteomics data have been deposited into the ProteomeXchange Consortium [54] (http://proteomecentral.proteomexchange.org) via the PRIDE partner repository [55] with the data set identifier PXD008885.

## Supplementary Materials

The following are available online at https://www.mdpi.com/article/10.3390/pathogens10091098/s1, Supplementary Dataset 1: Predicted surface proteins identified after “shaving” the living cells of the four *Streptococcus pneumoniae* strains in each of the seven culture conditions.
